# Correspondence: Quantitative evaluation of X-ray dark-field images for microcalcification analysis in mammography

**DOI:** 10.1038/ncomms10863

**Published:** 2016-04-22

**Authors:** Kai Scherer, Lorenz Birnbacher, Konstantin Willer, Michael Chabior, Julia Herzen, Franz Pfeiffer

**Affiliations:** 1 Lehrstuhl für Biomedizinische Physik, Physik-Department & Institut für Medizintechnik, Technische Universität München, 85748 Garching, Germany

Wang *et al.*^1^ have recently reported an X-ray grating interferometer imaging approach^2^,^3^,^4^ for mammography combining the information from the X-ray absorption and small-angle scattering signal. The authors claim that their approach can distinguish between type I (calcium oxalate dihydrate, CaC_2_O_4_·2H_2_O) and type II (calcium hydroxyapatite, Ca_5_(PO_4_)_3_(OH)) microcalcifications. While such a differentiation would indeed be of great value for clinical mammography, several important deficiencies in the study put the main results and conclusions of the published article in question. The shortcomings in the published work became obvious, after we have unsuccessfully tried to reproduce the results in our own laboratory.

To discriminate between type I and type II calcifications, Wang *et al.* use the ratio 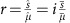 (equation (4) in Wang *et al.*^1^), where 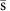 is the length-independent, effective X-ray scattering parameter and 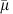 is the effective X-ray attenuation coefficient (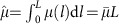, 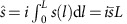, with *i* and *L* denoting a set-up-specific constant and the sample thickness, respectively). To test their hypothesis that type I and type II calcifications generally exhibit opposite absorption and scatter signals, they present (supposedly confirming) experimental results for a phantom made from calcium oxalate dehydrate and calcium hydroxyapatite powder (to mimic type I and type II calcifications, Fig. 1 in Wang *et al.*^1^). However, while the obtained values may be correct for the specific powders used here, the experimental outcome cannot be generalized easily, as the small-angle scattering signal does not only depend on the chemistry and density of the sample, but also strongly on the micromorphology of the powder. Previously published theoretical and experimental results^5^,^6^,^7^ clearly demonstrate this strong dependence of the scatter signal (and thus the *r*-value) on the average size of the microstructures. Consequently, arbitrarily chosen powders (with respect to the average grain size) cannot reliably model microcalcifications in the human breast, if the actual size distribution is not taken into account (and matched to the one in a real human breast). More specifically, our calculations (based on^6^) even show that by varying the average size of the powder microstructures, one can actually obtain arbitrary *r*-values, regardless of the actual chemical and density position. This is also reflected in a recent publication by Michel *et al.*^8^ which reports on a larger scattering signal in calcium oxalate dihydrate versus calcium hydroxyapatite, contradicting phantom results of Wang *et al.*^1^

Second, but probably even more important, we have identified a major mistake in the analysis of the data from the real breast specimens (Figs 3 and 5 in Wang *et al.*^1^), which render the main conclusions of the study highly questionable. In their evaluation of the *r*-value for various microcalcifications, Wang *et al.* have neglected the contribution of the underlying breast tissue. Correctly, the *r*-value has to be written as 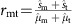, where the subscripts m and t denote contributions from the microcalcification and the tissue. While neglecting 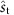 leads to a relatively small error in the *r*-value (as the scattering signal of tissue is relatively low), neglecting 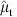 leads to a large error and significantly falsifies the classification of the microcalcifications. Some exemplary results from a corresponding experiment in our lab (Fig. 1) highlight the issue. The blue and the red points represent pixels with 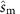
and 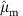 values of two different microcalcifications, and they appear as a cloud with a slope corresponding to the *r*_m_-value of this particular calcification. If now the contributions of the tissue (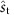 and 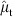) are neglected in the analysis, one obtains a slope (*r*-value) for the two clusters of *r*_1,Wang_=*r*_1,mt_=0.34±0.02 and *r*_2,Wang_=*r*_2,mt_=0.35±0.01, a very similar and small value in both cases (in agreement with Figs 3 and 5 in Wang *et al.*^1^). However, when the contributions from the tissue are now correctly subtracted, the real calcification values (matching the data cloud) become *r*_1,m_=6.63±0.18 and *r*_2,m_=2.48±0.07. This means that Wang *et al.*'s analysis would have yielded an error of almost 2000% for *r*_1_ and ∼700% for *r*_2_, with the consequence of large classification errors, as demonstrated by the example above (before correction: *r*_1_≈*r*_2_, after tissue correction: *r*_1_>>*r*_2_). Because of this error in the analysis, the data presented by Wang *et al.*^1^ can barely be associated with the calcifications themselves, but instead is mostly dominated by the attenuation of the breast tissue (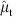
>>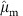), which renders a correct classification according to the hypothesis untenable. Accordingly, the presented *r*-values are small (0.3<*r*<1.0), whereas the real values obtained by a correct analysis show scatter dominated ratios (1.2<*r*<10).

In summary, we can conclude that the main claim of this article, namely the successful classification of different microcalcifications into type I and type II by this approach, is unjustified. Both, the experimental results of the phantom and the ones for the human breast samples, neglect major contributions to the image signal, and therefore render the main claim and specific experimental results and conclusions presented in this published study highly questionable.

Finally, we note that Wang *et al.* have neither discussed nor referenced related and partially contradicting, published results by other groups, in which detailed calculations and experimental verifications of the dependence of the scattering parameter on the sample microstructure are shown^5^,^6^,^7^,^8^. Furthermore, the authors have disregarded the fact that the use of the different ratios between attenuation and scattering parameters has already been demonstrated for material^9^ or tissue discrimination^10^.

## Additional information

**How to cite this article:** Scherer, K. *et al.* Correspondence: Quantitative evaluation of X-ray dark-field images for microcalcification analysis in mammography. *Nat. Commun.* 7:10863 doi: 10.1038/ncomms10863 (2016).

## Figures and Tables

**Figure 1:**
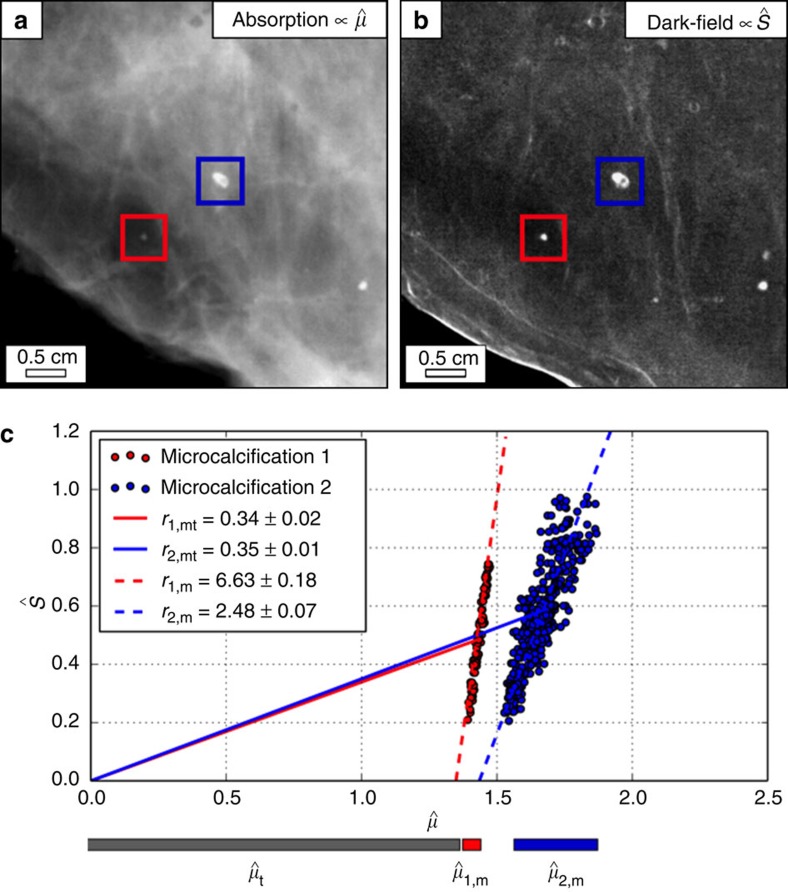
Quantitative evaluation of microcalcification analysis in X-ray dark-field mammography. (**a**) Experimental absorption and (**b**) dark-field mammogram of a freshly dissected breast abladate with microcalcifications. (**c**) Scatter plots comparing the absorption 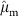 to scattering power 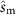 of two exemplary microcalcifications cluster, as indicated by the blue and red frame in (**a**, **b**), respectively. An incorrect *r*-value is obtained (*r*_Wang_=*r*_mt_) if contributions of the underlying tissue are neglected in the analysis, since 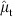
>>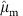.
